# Direct Marketing Promotion and Electronic Cigarette Use Among US Adults, National Adult Tobacco Survey, 2013–2014

**DOI:** 10.5888/pcd14.170073

**Published:** 2017-09-21

**Authors:** Hongying Dai, Jianqiang Hao

**Affiliations:** 1 Health Services and Outcomes Research, Children’s Mercy Hospital, Kansas City, Missouri; 2 Department of Biomedical and Health Informatics, University of Missouri-Kansas City, Kansas City, Missouri; 3 Department of Pediatrics, University of Missouri-Kansas City, Kansas City, Missouri; 4 Bellevue University, Omaha, Nebraska

## Abstract

**Introduction:**

The use of electronic cigarettes (e-cigarettes) among US adults has increased since 2007. The objective of this study was to investigate the prevalence of direct marketing promotion of e-cigarettes and its association with e-cigarette use among US adults.

**Methods:**

We used using data from the 2013–2014 National Adult Tobacco Survey (NATS) to estimate prevalence of e-cigarette promotions received by mail or email. Multinomial logistic regression was used to examine the associations between e-cigarette promotions and the prevalence and frequency of e-cigarette use among US adults.

**Results:**

In the 2013–2014 survey period, 7.1% of adults (about 16.0 million) reported receiving mail or email e-cigarette promotions in the previous 6 months; 3.2% received mail promotions, and 5.1% received email promotions. A higher prevalence of promotions was found among men versus women, adults aged under 65 years versus those older, current e-cigarette users, current smokers, and people with no smoking restriction rules in their homes or vehicles. In the multivariable analysis, receiving mail or email e-cigarette promotions was associated with higher odds of being current e-cigarette users (adjusted odds ratio [aOR] = 2.0; *P* < .001) than being noncurrent e-cigarette users. Receiving promotions was also associated with higher odds of using e-cigarettes some days (aOR = 1.6; *P* = .006) or every day (aOR = 1.7; *P* = .008) than using e-cigarettes rarely.

**Conclusion:**

Receipt of e-cigarette direct marketing promotions was prevalent among US adults. Receiving e-cigarette promotions was associated with increased odds of both prevalence and frequency of e-cigarette use. Future longitudinal studies are needed to measure causal effects of e-cigarette promotions on e-cigarette use among adults.

## Introduction

Although cigarette smoking among US adults declined significantly, from 42% in 1965 to 18% in 2012 (1), the use of electronic cigarettes (e-cigarettes) increased since 2007. From October 2013 through October 2014, 17.0% of US adults reported smoking cigarettes every day or some days, and 6.6% reported using e-cigarettes every day, some days, or rarely; from 2012 through 2013, 18.0% reported smoking cigarettes every day or some days, and 4.2% reported using e-cigarettes every day, some days, or rarely (2,3). Despite a rapid increase in e-cigarette use, their long-term health effects are unknown (4).

Cigarettes are one of the most heavily advertised products in the United States. The Federal Trade Commission reported that cigarette companies spent approximately $9.17 billion on cigarette advertising and promotion in 2012 (5). Studies have found that cigarette promotions were associated with increased smoking initiation, higher odds of smoking frequency and relapse, and reduced odds of quitting smoking (1). In recent years, e-cigarette makers have significantly increased their advertising expenditures (6). Exposure to e-cigarette advertisements and promotions is associated with increased use of e-cigarettes among youths and young adults (7–9).

Little is known about the association between the prevalence of direct marketing of e-cigarettes to adults and their increased use. Understanding this association is essential to countering the effects of these promotions. Furthermore, nearly half of current e-cigarette users reported using e-cigarettes rarely rather than every day or some days. (3) It is important to understand whether direct marketing promotions are associated with frequency of use. To address the gaps in knowledge, we used data from the 2013–2014 National Adult Tobacco Survey (NATS) to analyze the associations between direct marketing of e-cigarettes and the prevalence and frequency of e-cigarette use among US adults. This study sought to 1) estimate the prevalence of e-cigarette promotions by mail and email, both overall and stratified by sociodemographic characteristics, tobacco or e-cigarette use, and other related factors; 2) assess the association between e-cigarette promotions and prevalence of e-cigarette use; and 3) examine the association between e-cigarette promotions and frequency of e-cigarette use.

## Methods

### Data

NATS was conducted from October 2013 through October 2014 among a sample of adults aged 18 years or older in the 50 US states and the District of Columbia. The purpose of the survey was to determine the prevalence of tobacco use among adults and the factors promoting and impeding its use. The survey consisted of 75,233 qualified interviews from either landline or cellular telephones. The overall response rate was 36.1%. The landline response rate was 47.6%, and the cellular telephone response rate was 17.1%. A detailed description of the NATS design, questionnaires, and data collection are available (10). National weights and strata were applied to each record to account for the complex survey design and to adjust for nonresponse. Because data were de-identified and publicly available, the institutional review board of Children’s Mercy Hospital considered the study not human subjects research.

### Measures

Three questions from NATS were used to define e-cigarette use: 1) “Before today, had you ever heard of electronic cigarettes or e-cigarettes?” with response options of yes or no; 2) “Have you ever used an electronic cigarette, even just one time, in your entire life?” with response options of yes or no; and 3) “Do you now use electronic cigarettes?” with response options of “every day,” “some days,” “rarely,” or “not at all.” We classified survey respondents as current e-cigarette users (people who reported using e-cigarettes every day, some days, or rarely) or noncurrent e-cigarette users (people who had never heard of e-cigarettes, had never tried e-cigarettes, or did not currently use e-cigarettes).

E-cigarette promotions through mail or email were measured by the following 2 questions from NATS: 1) “In the past 6 months, have you received any mail addressed to you from a company that manufactures e-cigarettes?” and 2) “In the past 6 months, have you received any email from a company that manufactures e-cigarettes?” Respondents who answered yes to the first question were classified as “receive mail promotion” (code = 1), those who answered yes to the second question were classified as “receive email promotion” (code = 1), and those who answered yes to either of these 2 questions were classified as “receive mail or email promotion” (code = 1). Those who answered no were coded as 0. Because mail and email promotions could overlap, we created a mutually exclusive variable to assess the additive effect of multiple promotions: no promotion (code = 0), single promotion (either mail or email promotions but not both, code = 1), and both mail and email promotions (code = 2).

Several covariates were included in the analysis to control for confounding effects, such as age (18–29 y, 30–39 y, 40–49 y, 50–64 y, ≥65 y), sex (male or female), race/ethnicity (non-Hispanic white, non-Hispanic black, Hispanic, or other), education (less than high school diploma, high school diploma or general equivalency degree, some college, and bachelor’s degree or higher) and annual household income (0–$29,999, $30,000–$49,999, $50,000–$99,999, ≥$100,000). Because there is a high positive correlation between e-cigarette use and traditional cigarette use among adults (11,12), we included current tobacco smoking as a covariate. Current tobacco smoking status was coded as never smokers (respondents who answered no to smoking at least 100 cigarettes in their entire life), former smokers (respondents who reported smoking at least 100 cigarettes in their entire life and reported not currently smoking at all), and current smokers (respondents who reported smoking at least 100 cigarettes in their entire life and currently smoking every day or some days).

Smoking rules were also included as covariates in the analyses. Having a home smoking rule was measured by the question “Not counting decks, porches, or garages, inside your home, is smoking . . . ?” The responses were classified into full home smoking rule (never allowed), partial home smoking rule (allowed only at some times or in some places), and no home smoking rule (always allowed). Having a vehicle smoking rule was measured by the question “Not counting motorcycles, in the vehicles that you or family members who live with you own or lease, is smoking . . . ?” The responses were classified into full vehicle smoking rule (never allowed in any vehicle), partial vehicle smoking rule (sometimes allowed in at least one vehicle), and no vehicle smoking rule (always allowed).

Housing type has been found to be associated with the use of tobacco products, including e-cigarettes (13); therefore, this variable was included as a covariate in the multivariable analysis. Housing types were classified as single-family house, multiunit house, and “other” based on responses to the question from 2013–2014 NATS, “In what type of living space do you currently reside?”

In addition, because people might intentionally sign up for promotions from e-cigarette manufacturers, we added this type of proactive behavior as a covariate on the basis of this NATS question: “Ever intentionally submitted your mailing address to sign up for offers or promotions from a company that manufactures e-cigarettes?” Those who responded yes were classified as “submit mail address” (code = 1) and those who responded no were coded as 0.

### Statistical analysis

Weighted estimates of prevalence of e-cigarette promotions (mail, email, or either) were calculated, both overall and stratified by sociodemographic characteristic, cigarette or e-cigarette use status, smoking rules (home or vehicle), and housing type. Logistic regression models were used to evaluate the associations between e-cigarette promotions (mail, email, either, both) and prevalence of e-cigarette use; multinomial logistic regression models were used to evaluate the associations between e-cigarette promotions and frequency of e-cigarette use. To assess the additive effect of multiple promotions, people receiving both email and mail promotions were compared with those receiving a single promotion (either email or mail but not both). Adjusted odds ratios (aORs) were calculated in the multivariable analysis in which all promotion variables and covariates were included as explanatory variables. Those who reported not currently using e-cigarettes served as the control group in analyzing the prevalence of e-cigarette use, and those who reported rarely using e-cigarettes served as the control group in analyzing the frequency of e-cigarette use. The prevalence of promotions was calculated by using SURVEYFREQ, SAS 9.4 (SAS Institute, Inc) and the difference in promotion prevalence was tested by Rao–Scott χ^2^ test (14). The associations between promotion and e-cigarette use were analyzed by using SURVEYLOGISTIC, SAS 9.4. Sampling weight and survey strata were included in the analysis. We used SAS 9.4 to perform all statistical analyses. A *P* value of less than .05 was considered significant.

## Results

In NATS, 7.1% of adults (about 16.0 million) reported receiving mail or email e-cigarette promotions in the 6 months before the survey, including 3.2% (about 7.1 million) who received mail promotions and 5.1% (about 11.3 million) who received email promotions (Tables 1 and 2). Of the 7.1 million who received mail promotions, 5.8 million (81%) were not current e-cigarette users. Of 11.3 million who received email promotions, 9 million (80%) were not current e-cigarette users. In addition, the majority of promotions were sent to people who may not have signed up intentionally for the promotions. For instance, 76% of recipients (5.4 million) of mail promotions did not intentionally submit their mail addresses. Adults aged 30 to 39 years had the highest prevalence of receiving e-cigarette promotions by mail (4.3%), and those aged 40 to 49 years had the highest prevalence of receiving email e-cigarette promotions (6.3%). Adults aged 30 to 49 years were more likely to receive mail or email promotions (8.4%) from e-cigarette manufactures than those aged 18 to 29 (6.7%) or those aged 65 years or older (4.1%). Adults with some college had the highest prevalence of receiving mail promotions (3.9%) or email promotions (6.6%). Adults with some college had the highest prevalence of receiving mail or email promotions (9.0%), and people with annual incomes less than $30,000 reported the lowest prevalence of receiving mail or email promotions (6.6%). Current smokers and current e-cigarette users reported the highest prevalence of receiving mail or email promotions. The prevalence of receiving e-cigarette promotions was positively correlated with smoking rules; the lowest prevalence was among people with full home smoking rules or full vehicle smoking rules. As expected, those who intentionally submitted their mail addresses to e-cigarette manufacturers had a much higher likelihood of receiving mail or email promotions than those who did not.

**Table 1:** Prevalence of e-Cigarette Promotions by Mail and Email Received in Previous 6 Months, Overall and by Sociodemographic and Smoking Factors, Among US Adults, National Adult Tobacco Survey, 2013–2014^a^

Variable	Mail Promotion			Email Promotion		
	n	Weighted n, in Thousands	Prevalence (95% CI)	n	Weighted n, in Thousands	Prevalence (95% CI)
**Overall**	1,960	7,120	3.2 (3.0–3.4)	3,422	11,250	5.1 (4.9–5.4)
**Sex**	*P* < .001			*P* < .001		
Male	1,010	3,960	3.7 (3.4–4.0)	1,662	5,950	5.6 (5.3–6.0)
Female	946	3,150	2.8 (2.5–3.0)	1,748	5,270	4.7 (4.4–4.9)
**Age, y**	*P* < .001			*P* < .001		
18–29	245	1,390	3.1 (2.6–3.5)	381	2,060	4.7 (4.1–5.2)
30–39	307	1,650	4.3 (3.7–4.9)	481	2,230	5.9 (5.2–6.5)
40–49	295	1,310	3.6 (3.1–4.1)	570	2,260	6.3 (5.7–6.9)
50–64	610	1,880	3.2 (2.9–3.6)	1,303	3,550	6.1 (5.7–6.5)
≥65	486	850	2.1 (1.9–2.3)	661	1,070	2.7 (2.4–2.9)
**Race/ethnicity**	*P* = .15			*P* < .001		
Non-Hispanic white	1,423	4,540	3.2 (2.9–3.4)	2,619	7,660	5.3 (5.1–5.6)
Non-Hispanic black	217	1,000	3.9 (3.3–4.5)	287	1,210	4.8 (4.1–5.5)
Hispanic	154	970	3.1 (2.5–3.7)	214	1,150	3.7 (3.1–4.3)
Other	145	550	3.2 (2.5–3.9)	267	1,140	6.7 (5.7–7.8)
**Education**	*P* < .001			*P* < .001		
Less than high school diploma	134	680	2.4 (1.9–2.9)	112	630	2.3 (1.7–2.8)
High school diploma or GED	419	2,020	3.3 (2.9–3.7)	579	2,630	4.3 (3.9–4.7)
Some college	677	2,680	3.9 (3.6–4.3)	1,208	4,480	6.6 (6.2–7.1)
Bachelor’s degree or higher	714	1,690	2.8 (2.5–3.0)	1,503	3,450	5.7 (5.3–6.0)
**Annual household income, $**	*P* = .006			*P* < .001		
0–29,999	355	1,210	3.3 (2.8–3.7)	445	1,590	4.3 (3.8–4.9)
30,000–49,999	419	1,650	4.2 (3.7–4.7)	625	2,110	5.4 (4.8–5.9)
50,000–99,999	521	1,880	3.4 (3.1–3.8)	1,044	3,270	6.0 (5.5–6.5)
≥100,000	345	1,160	3.1 (2.7–3.5)	794	2,500	6.6 (6.0–7.2)
**Cigarette smoking**	*P* < .001			*P* < .001		
Never	764	2,650	2.1 (1.9–2.3)	1,533	4,870	3.9 (3.6–4.1)
Former	645	2,020	3.6 (3.3–4.0)	1,112	3,400	6.1 (5.6–6.6)
Current	545	2,440	6.4 (5.7–7.1)	760	2,930	7.9 (7.1–8.6)
**Current e-cigarette use**	*P* < .001			*P* < .001		
No	1,657	5,770	2.8 (2.6–3.0)	2,869	9,000	4.4 (4.2–4.6)
Yes	298	1,340	9 (7.7–10.2)	541	2,220	15.1 (13.5–16.6)
**Home smoking rule**	*P* < .001			*P* < .001		
Full	1,454	5,160	2.9 (2.7–3.1)	2,690	8,570	4.8 (4.6–5.1)
Partial	205	740	3.9 (3.2–4.5)	328	1,210	6.4 (5.5–7.2)
None	211	840	5.8 (4.8–6.9)	315	1,160	8.1 (7.0–9.3)
**Vehicle smoking rule**	*P* < .001			*P* < .001		
Full	1,260	4,230	2.6 (2.4–2.7)	2,452	7,710	4.6 (4.4–4.9)
Partial	356	1,410	5.1 (4.4–5.7)	545	2,010	7.3 (6.5–8.1)
None	239	1,070	6.9 (5.7–8.0)	322	1,170	7.6 (6.5–8.8)
**Housing type**	*P* = .69			*P* = .22		
Single family	1,357	4,720	3.3 (3.1–3.5)	2,434	7,420	5.2 (4.9–5.4)
Multiunit	443	1,770	3.1 (2.7–3.5)	753	3,030	5.3 (4.8–5.9)
Other	137	510	3.3 (2.6–4.0)	202	680	4.5 (3.7–5.3)
**Submitted mailing address**	*P* < .001			*P* < .001		
No	1,562	5,390	2.5 (2.4–2.7)	2,897	9,260	4.4 (4.2–4.6)
Yes	383	1,680	27.0 (24.0–30.0)	487	1,840	29.9 (27.0–32.9)

Abbreviations: CI, confidence interval; GED, general equivalency degree.

a Number estimates were weighted by taking sample weight and strata into account. Weighted total number of users is rounded to the nearest 10,000. Significance was set at *P* < .05. *P* values of the difference in promotion prevalence were calculated by using Rao-Scott χ^2^ test.

**Table 2:** Prevalence of e-Cigarette Promotions, by Mail or Email and Both Mail and Email, Received in Previous 6 Months, Overall and by Sociodemographic and Smoking Factors, Among US Adults, National Adult Tobacco Survey, 2013–2014^a^

Variable	Mail or Email Promotion			Both Mail and Email Promotion		
	n	Weighted n, in thousands	Prevalence (95% CI)	n	Weighted n, in thousands	Prevalence (95% CI)
**Overall**	4,709	16,020	7.1 (6.9–7.4)	673	2,360	1.1 (0.9–1.2)
**Sex**	*P* < .001			*P* < .001		
Male	2,322	8,600	8.0 (7.6–8.4)	350	1,310	1.2 (1.0–1.4)
Female	2,373	7,380	6.4 (6.0–6.7)	321	1,040	0.9 (0.8–1.0)
**Age**	*P* < .001			*P* < .001		
18–29	544	3,030	6.7 (6.1–7.4)	82	420	0.9 (0.7–1.2)
30–39	675	3,300	8.4 (7.6–9.2)	113	580	1.5 (1.1–1.8)
40–49	754	3,120	8.4 (7.7–9.1)	111	460	1.2 (1.0-1.5)
50–64	1,695	4,760	8.0 (7.5–8.5)	218	660	1.1 (0.9–1.3)
≥65	1	1,690	4.1 (3.8–4.4)	147	240	0.6 (0.5–0.7)
**Race/ethnicity**	*P* = .003			*P* < .001		
Non-Hispanic white	3,563	10,750	7.3 (7.0–7.6)	479	1,440	1.0 (0.9–1.1)
Non-Hispanic black	432	1,920	7.4 (6.6–8.3)	72	280	1.1 (0.8–1.4)
Hispanic	317	1,790	5.6 (4.8–6.4)	51	340	1.1 (0.7–1.4)
Other	346	1,400	8.0 (6.9–9.1)	66	290	1.7 (1.2–2.2)
**Education**	*P* < .001			*P* < .001		
Less than high school diploma	220	1,170	4.1 (3.4–4.8)	26	140	0.5 (0.2–0.8)
High school diploma or GED	867	4,040	6.5 (6.0–7.0)	131	610	1.0 (0.8–1.2)
Some college	1,652	6,220	9.0 (8.4–9.5)	233	940	1.4 (1.1–1.6)
Bachelor’s degree or higher	1,940	4,480	7.2 (6.8–7.6)	277	660	1.0 (0.9–1.2)
**Annual household income, $**	*P* = .001			*P* = .01		
0-29,999	702	2,450	6.6 (6.0–7.3)	98	340	0.9 (0.7–1.2)
30,000–49,999	908	3,290	8.2 (7.5–8.9)	136	470	1.2 (0.9–1.4)
50,000–99,999	1,370	4,520	8.1 (7.6–8.7)	195	630	1.1 (0.9–1.3)
≥100,000	1,007	3,170	8.2 (7.6–8.8)	132	480	1.3 (1.0-1.5)
**Cigarette smoking**	*P* < .001			*P* < .0001		
Never	2,073	6,720	5.2 (5.0–5.5)	224	810	0.6 (0.5–0.7)
Former	1,512	4,670	8.2 (7.7–8.7)	245	750	1.3 (1.1–1.5)
Current	1,103	4,570	12.0 (11.1–12.9)	202	810	2.1 (1.7–2.5)
**Current e-cigarette use**	*P* < .001			*P* < .001		
No	4,029	13,090	6.3 (6.0-6.5)	497	1,670	0.8 (0.7–0.9)
Yes	665	2,880	19.2 (17.5–20.9)	174	680	4.6 (3.7–5.4)
**Home smoking rule**	*P* < .001			*P* < .001		
Full	3,651	12,010	6.6 (6.4–6.9)	493	1,710	0.9 (0.8–1.1)
Partial	463	1,720	8.9 (7.9–9.9)	70	230	1.2 (0.9–1.5)
None	446	1,700	11.7 (10.3–13.1)	80	300	2.1 (1.5–2.7)
**Vehicle smoking rule**	*P* < .001			*P* < .001		
Full	3,281	10,460	6.2 (5.9–6.5)	431	1,470	0.9 (0.8–1.0)
Partial	773	2,990	10.6 (9.7–11.6)	128	430	1.5 (1.2–1.9)
None	481	1,910	12.2 (10.8–13.6)	80	330	2.1 (1.5–2.7)
**Housing type**	*P* = .57			*P* = .66		
Single family	3,331	10,650	7.2 (6.9–7.6)	460	1,490	1.0 (0.9–1.1)
Multiunit	1,036	4,140	7.2 (6.6–7.7)	160	660	1.1 (0.9–1.4)
Other	297	1,040	6.7 (5.7–7.6)	42	150	1.0 (0.6–1.3)
**Submitted mailing address**	*P* < .001			*P* < .001		
No	4	13,130	6.1 (5.8–6.3)	459	1,520	0.7 (0.6–0.8)
Yes	664	2,710	43.1 (39.9–46.4)	206	810	12.9 (10.8–15.0)

Abbreviations: CI, confidence interval; GED, general equivalency degree.

a Number estimates were weighted by taking sample weight and strata into account. Weighted total number of users is rounded to the nearest 10,000. Significance was set at *P* < .05. *P* values of the difference in promotion prevalence were calculated by using Rao-Scott χ^2^ test.

After adjusting for all covariates, such as sociodemographic factors, smoking status, smoking rules, housing type, and whether respondents intentionally submitted their mail addresses, receiving mail promotions was not associated with increased odds of being a current e-cigarette user (aOR, 1.2; *P* = .09) (Table 3). Compared with those who did not receive email promotions, adults who received email promotions in the 6 months before 2013–2014 NATS had higher adjusted odds of being a current e-cigarette user (aOR, 2.6; *P* < .001). Overall, receiving mail or email e-cigarette promotions significantly increased the odds of being a current e-cigarette user (aOR, 2.0; *P* < .001). Receiving both mail and email e-cigarette promotions was not associated with greater odds of being a current e-cigarette user than receiving a single promotion (either mail or email, not both) (aOR, 1.2; *P* = .31).

**Table 3:** Association Between Prevalence of e-Cigarette Use and Tobacco Company Promotions Received in Previous 6 Months Among US Adults Who Were Current Users, National Adult Tobacco Survey, 2013–2014^a^

Variable	Current E-Cigarette Use	
	Adjusted Odds Ratio (95% Confidence Interval)	*P* Value
**Mail promotion**		
No	1 [Reference]	
Yes	1.2 (1.0–1.6)	.09
**Email promotion**		
No	1 [Reference]	
Yes	2.6 (2.1–3.1)	<.001
**Mail or email promotion**		
No	1 [Reference]	
Yes	2.0 (1.7–2.4)	<.001
**Mail and email promotion**		
Single promotion	1 [Reference]	
Both promotions	1.2 (0.8–1.7)	.31

a Adjusted odds ratios for current e-cigarette use are in reference to non-current e-cigarette use. Adjusted odds ratios are adjusted by all covariates in the study, including sex, age, race/ethnicity, education, income, smoking rules, housing unit type, and whether respondents intentionally submitted their mail addresses to e-cigarette manufacturers.

After adjusting for other covariates, receiving mail promotions was associated with increased odds of using e-cigarettes every day (aOR, 1.7; *P* = .04); receiving email promotions was associated with increased odds of using e-cigarettes every day (aOR, 2.0; *P* = .001 and some days (aOR, 1.5; *P* = .03) (Table 4). Overall, receiving mail or email e-cigarette promotions significantly increased odds of using e-cigarettes every day (aOR, 1.7; *P* = .008) and some days (aOR, 1.6; *P* = .006). People who received both mail and email e-cigarette promotions had higher odds of using e-cigarettes every day than those who received only a single promotion (aOR, 3.1; *P* = .003).

**Table 4:** Association Between Frequency of E-Cigarette Use and Tobacco Company Promotions Received in Previous 6 Months Among US Adults Who Were Current Users, National Adult Tobacco Survey, 2013–2014^a^

Variable	Frequency			
	Some Days		Every Day	
	Adjusted Odds Ratio (95% Confidence Interval)	*P* Value	Adjusted Odds Ratio (95% Confidence Interval)	*P* Value
**Mail promotion**				
No	1 [Reference]		1 [Reference]	
Yes	1.5 (1.0-2.3)	.07	1.7 (1.0–2.7)	.04
**Email promotion**				
No	1 [Reference]		1 [Reference]	
Yes	1.5 (1.1–2.2)	.03	2.0 (1.4–3.0)	<.001
**Mail or email promotion**				
No	1 [Reference]		1 [Reference]	
Yes	1.6 (1.1–2.2)	.006	1.7 (1.2–2.4)	.008
**Mail and email promotion**				
Single promotion	1 [Reference]		1 [Reference]	
Both promotions	1.2 (0.6–2.5)	.56	3.1 (1.5–6.6)	.003

a Adjusted odds ratios for use of e-cigarettes on some days and every day are in reference to “rarely” use e-cigarettes. Adjusted odds ratios are adjusted by all covariates in the study, including sex, age, race/ethnicity, education, income, smoking rules, housing unit type, and whether respondents intentionally submitted their mail addresses to e-cigarette manufacturers.

## Discussion

Tobacco marketing is causally related to tobacco use (15), and e-cigarette use has gained popularity since it entered the US market in 2007. We found that direct marketing promotion of e-cigarettes to US adults was prevalent. As expected, current smokers and e-cigarette users were more likely to receive mail and email promotions. However, of greatest concern is that a large percentage of these promotions were sent to people who had never smoked or used e-cigarettes. In addition, most promotions were sent to people who said they did not sign up intentionally for the promotions. These people may have been added to direct marketing databases from online searches, social networks, or purchases of e-cigarettes, which could make current and former e-cigarette users more likely to receive promotions.

Tobacco companies have been using direct marketing, including mail, web, email, and mobile marketing platforms, to recruit new customers, retain existing customers, and build loyalty. In 2013, US cigarette and smokeless tobacco companies spent $68.8 million in direct marketing and an additional $281.1 million on coupons, which were often distributed through direct marketing channels (16,17). The 2009 Family Smoking Prevention and Tobacco Control Act enables the US Food and Drug Administration (FDA) to regulate tobacco sales, distribution, and accessibility and the advertising and promotion of tobacco products. In May 2016, FDA announced final rules to extend its regulatory authority over e-cigarettes and other newly deemed tobacco products along with other restrictions (18). These regulations require inclusion of nicotine addiction warning statements on e-cigarette advertisements (19). Because e-cigarette makers may have adopted direct marketing strategies similar to those of tobacco companies, educational campaigns and antivaping commercials may help educate the general public about potential health risks of e-cigarette use (20,21).

Tobacco companies use direct marketing to target vulnerable populations, such as young adults, women, and people of low socioeconomic status (22–24). This study adds to existing literature by extending our understanding of the heterogeneity in e-cigarette direct marketing. For instance, adults aged 30 to 49 years were more likely to receive mail or email promotions from e-cigarette manufacturers than those in younger or older age groups we studied. Adults with some college had the highest prevalence of receiving mail or email promotions of the groups studied, and those with annual income less than $30,000 had the lowest prevalence. These inequities in mail or email promotions are not completely aligned with current e-cigarette use, which was correlated with being a current smoker, a young adult, of lower income, of lower education, and non-Hispanic white (3,25). Our results suggest that e-cigarette manufactures may be using direct marketing as a tool to expand their customer base, reach new customers, and build a loyal customer base. Continuous monitoring of e-cigarette direct marketing is warranted to prevent the targeting of vulnerable populations with mail or email promotions.

Consistent with the findings from previous studies that tobacco marketing is associated with increased risk of cigarette use and smoking frequency (1,26,27), our cross-sectional study confirmed that e-cigarette promotions were associated with e-cigarette use (current use and frequency of use) among US adults. Although e-cigarettes are generally less harmful than traditional combustible cigarettes, they contain varying levels of nicotine and numerous potentially toxic substances, including some known or suspected carcinogens (4,28). The long-term health effects of e-cigarette use are unclear, and whether e-cigarettes could be effective in smoking cessation is still controversial (29). Tobacco control strategies could be developed to counteract the effects from e-cigarette marketing and promotions.

This study has limitations. First, NATS data are cross-sectional. Though we attempted to adjust for other covariates — sociodemographic factors, smoking status, smoking rules, housing type, and whether respondents intentionally submitted their addresses for e-cigarette promotions — residual confounding effects by other covariates could explain some associations. Therefore, we were unable to establish a causal relationship between receiving e-cigarette promotions and increased use of e-cigarettes. Second, self-report of e-cigarette and tobacco use might lead to misreporting. However, self-reported cigarette smoking correlated highly with measured serum cotinine levels among adults (30). Third, receiving e-cigarette promotions was self-reported. Thus, recall and attentional biases might exist in this study, because current e-cigarette users and those using e-cigarettes every day or some days might be more likely to pay attention to e-cigarette promotions than those who are not interested in e-cigarettes. Fourth, this study does not address online promotions (eg, web, social media), which have increased in recent years. Thus, it is likely that the study underestimates the total amount of e-cigarette promotion survey respondents were exposed to. Finally, the overall response rate was low at 36.1% and could result in bias, even after adjustment for nonresponse.

Despite the limitations, this study identified that about 16 million US adults received mail or email e-cigarette marketing promotions during the period covered by NATS. The results of this cross-sectional study indicate that receiving e-cigarette promotions was associated with increased odds of both prevalence and frequency of e-cigarette use. Future longitudinal studies are needed to measure the causal effects of e-cigarette promotions on e-cigarette use among adults.
